# Detection of the early stage of spontaneous hemopneumothorax by CT attenuation values

**DOI:** 10.1002/ccr3.2849

**Published:** 2020-04-20

**Authors:** Yukiko Koike, Ho Namkoong, Sotaro Otake, Hisao Asamura

**Affiliations:** ^1^ Department of Pulmonary Medicine Eiju General Hospital Tokyo Japan; ^2^ Laboratory of Clinical Immunology and Microbiology National Institute of Allergy and Infectious Diseases NIH Bethesda MD USA; ^3^ Division of Thoracic Surgery Keio University School of Medicine Tokyo Japan

**Keywords:** CT attenuation values, hemopneumothorax

## Abstract

Spontaneous hemopneumothorax is potentially fatal and can mimic spontaneous pneumothorax when changes in vital signs and bloody discharge from the chest tube are absent. Attenuation values at computed tomography may help detect this condition.

## Graphical abstract

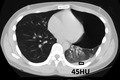

A 21‐year‐old man presented with acute chest pain and dyspnea. On admission, his vital signs were normal. Chest radiography showed left lung collapse, and chest computed tomography (CT) displayed slight pleural effusion with pneumothorax (Figure 1A,B). A chest tube was inserted without complications. Six hours after insertion, he suddenly complained of general malaise, and his vital signs indicated a shock. Hemopneumothorax was diagnosed after spontaneous discharge of 500 mL of blood from the chest tube. Chest CT revealed rapid increase in pleural effusion (Figure 1C). Video‐assisted thoracoscopic surgery was emergently performed. Formation of new blood vessels between the parietal and visceral pleura was identified as the source of bleeding, which was located adjacent to ruptured bullae (Figure 1D,E). The postoperative course was uneventful.

**Figure 1:**
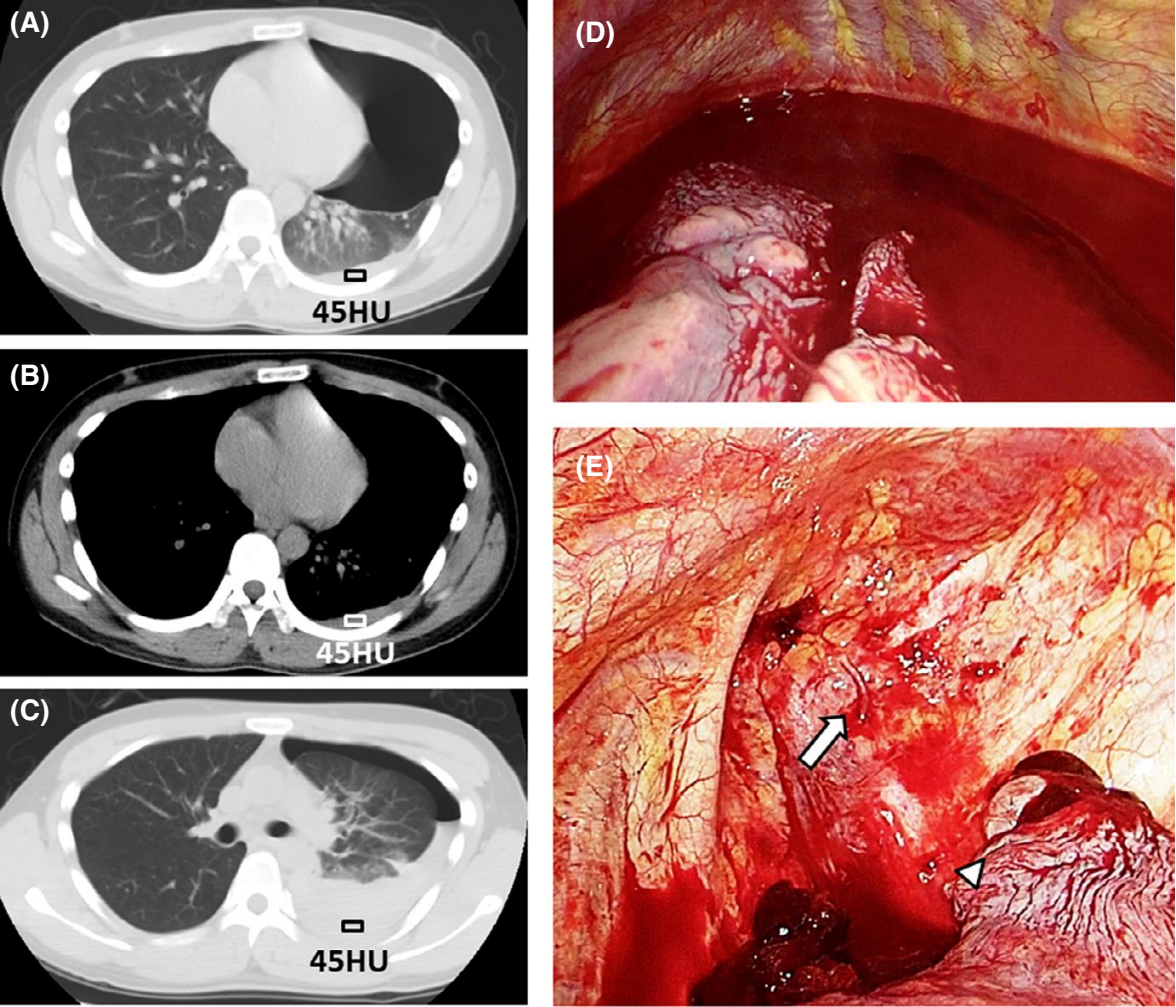
Chest CT on admission showing a tiny amount of pleural effusion with pneumothorax. The CT attenuation values of pleural effusion at this time were 45 Hounsfield Units, indicating that the pleural effusion was blood. (A: lung window, B: mediastinal window). Chest CT at the time of shock status showing rapid increase in pleural effusion. (C: lung window), (D) Intraoperative findings of video‐assisted thoracoscopic surgery showing accumulation of massive blood within the pleural space. (E) Intraoperative findings of video‐assisted thoracoscopic surgery showing that the formation of new blood vessels between the parietal and visceral pleura was the source of bleeding (arrow). The source of bleeding was located adjacent to ruptured bullae (triangle). CT, computed tomography; HU, Hounsefield Units

Spontaneous hemopneumothorax shows accumulation of blood within the pleural space without trauma or other causes.^1^ Retrospective analysis of CT attenuation values (Figure 1A,B), which showed 45 Hounsfield units, suggested the presence of blood. Therefore, careful analysis of CT attenuation values is useful to detect very early stages of spontaneous hemopneumothorax especially when it exceeds 35HU.^2^ Physicians and radiologists should thoroughly evaluate attenuation values even with normal vital signs and in the absence of bloody drainage from chest tubes to detect hemopneumothorax mimicking spontaneous pneumothorax before progression.

## CONFLICT OF INTEREST

The authors have declared that no competing interests exist.

Informed consent: Written informed consent was obtained from the patient.

## AUTHOR CONTRIBUTION

KY: involved in patient care and writing‐original draft preparation. HN: involved in patient care, investigation, and writing‐original draft preparation. SO: involved in patient care and editing. HA: involved in patient care and editing.
